# Minimal domain peptides derived from enterocins exhibit potent antifungal activity

**DOI:** 10.3389/ffunb.2024.1506315

**Published:** 2024-12-19

**Authors:** Dorrian G. Cohen, Theresa M. Heidenreich, Jason W. Schorey, Jessica N. Ross, Daniel E. Hammers, Henry M. Vu, Thomas E. Moran, Christopher J. Winski, Peter V. Stuckey, Robbi L. Ross, Elizabeth Arsenault Yee, Felipe H. Santiago-Tirado, Shaun W. Lee

**Affiliations:** ^1^ Department of Biological Sciences, University of Notre Dame, Notre Dame, IN, United States; ^2^ Eck Institute for Global Health, Notre Dame, IN, United States

**Keywords:** fungi, *Cryptococcus neoformans*, fungal infections, synthetic peptides, antimicrobial peptides, AS-48, bacteriocins, rational design

## Abstract

The antimicrobial peptide (AMP) circularized bacteriocin enterocin AS-48 produced by *Enterococcus* sp. exhibits broad-spectrum antibacterial activity via dimer insertion into the plasma membrane to form membrane pore structures, compromising membrane integrity and leading to bactericidal activity. A specific alpha-helical region of enterocin AS-48 has been shown to be responsible for the membrane-penetrating activity of the peptide. The canon syn-enterocin peptide library, generated using rational design techniques to have ninety-five synthetic peptide variants from the truncated, linearized, membrane-interacting domain of enterocin AS-48, was screened against three clinically relevant fungal strains: *Cryptococcus neoformans*, *Candida albicans*, and *Candida auris* for potential antifungal activity. Twelve peptides exhibited antifungal activity against *C. neoformans*, and two peptides exhibited activity against *C. albicans*. The fourteen active antifungal peptides were minimally cytotoxic to an immortalized human keratinocyte cell line (HaCats). Four select peptides were identified with minimum inhibitory concentrations (MICs) below 8 µM against *C. neoformans*. In 36-hour cell growth tests with these fungicidal peptides, fungicidal peptide no. 32 displayed inhibitory properties comparable to the leading antifungal medication fluconazole against *C. neoformans*. Screening of peptide no. 32 against a deletion library of *C. neoformans* mutants revealed that the mechanism of action of peptide no. 32 may relate to multivesicular bodies (MVBs) or polysaccharide capsule targeting. These findings importantly demonstrate that naturally derived AMPs produced by bacteria can be sourced, engineered, and modified to exhibit potent antifungal activity. Our results will contribute to the development of broad treatment alternatives to fungal infections and lend themselves to direct implications for possible treatment options for *C. neoformans* infections.

## Introduction

Each year, severe fungal infections afflict over 150 million people worldwide, resulting in over 1.7 million deaths (Denning, 2024; Bongomin et al., 2017; Kainz et al., 2020). Infection rates have been rapidly increasing as social and medical factors such as those associated with COVID-19 cause escalated fungal infections (Bongomin et al., 2017; Basile et al., 2022; Hoenigl et al., 2022; Mina et al., 2022; Hlaing et al., 2023). Only three classes of antifungal drugs are currently used clinically against fungal infections: polyenes such as amphotericin B, azoles such as fluconazole, and echinocandins such as caspofungin (Revie et al., 2018; Berman and Krysan, 2020; Lee et al., 2021). Widespread use of antifungal drugs has led to numerous fungal strains developing resistances to these traditional antifungal agents, and the loss of efficacy of even one class of antifungal drugs against a fungal infection removes one third of treatment options if all three classes were initially effective against that infection (Berman and Krysan, 2020). Drug target alteration, drug target overexpression, multidrug transporter upregulation, and stress response activation are only four of the adaptive mechanisms by which fungi, such as those within the genera *Cryptococcus* and *Candida*, have developed resistance to antifungal drugs (Shapiro et al., 2011; Revie et al., 2018). Innovative methods of treating fungal infections must be explored in order to fill the growing gaps in the antifungal drug development pipeline.

While there are several dozen fungal species that can cause disease in humans, two of the most common disease-causing fungal genera are *Cryptococcus* and *Candida* (Schmiedel and Zimmerli, 2016; Strickland and Shi, 2021). Annually, *Cryptococcus neoformans* causes approximately 220,000 cases of cryptococcal meningitis worldwide and 181,000 deaths concentrated in sub-Saharan Africa (Rajasingham et al., 2017; Rodrigues and Nosanchuk, 2020; Rajasingham et al., 2022). In light of the great public health threat that *C. neoformans* presents and the limited resources being dedicated to its research, *C. neoformans* was named a Critical Priority Group in the first-ever WHO fungal priority pathogens list (WHO FPPL) in October 2022, along with *Candida auris* and *Candida albicans* (WHO fungal priority pathogens list). *Candida auris* infections have become a major concern in the last thirteen years, as multidrug resistant strains rapidly spread across the United States and the world (Hu et al., 2021). *C. auris* isolates that are resistant to staple antifungal drugs such as fluconazole and amphotericin B are common, while isolates resistant to echinocandins are rarer (Du et al., 2020). Multidrug resistant *C. auris* is associated with an increased mortality rate; thus, novel antifungals are of immediate need for these infections.

Antimicrobial peptides (AMPs) are one innovative approach to producing antifungal and other antimicrobial treatments. AMPs are a class of small peptides that have variable inhibitory or selective cytotoxic effects on bacteria, viruses, and fungi as well as having anti-inflammatory and antibiofilm effects (Luo and Song, 2021; Moretta et al., 2021). Over 60 AMP drugs are already available commercially, with hundreds more AMPs in clinical or preclinical development (Boparai and Sharma, 2019). For example, the antiviral peptide Fuzeon™ is used as an anti-HIV drug, inhibiting HIV entry by targeting one site within the viral transmembrane envelope protein (Ashkenazi et al., 2011; Huan et al., 2020). The design of novel AMPs has also extended to diverse antifungal applications. Two chemically synthesized radish yeasts Rs-AFP1 and Rs-AFP2 producing AMPs have shown effective inhibition against food spoilage yeast such as *Zygosaccharomyces bailii* and *Zygosaccharomyces rouxii* (Huan et al., 2020; Shwaiki et al., 2020). The heptapeptide AurH1 was designed from Aurein1.2, an AMP secreted by the Australian tree frog *Litoria aurea*, and has shown specific and effective treatment of *C. albicans* infections (Madanchi et al., 2019; Zheng et al., 2021).

Bacteriocins are a large class of ribosomally synthesized peptides produced by bacteria. Nisin, likely the best-known bacteriocin-derived AMP, is an FDA-approved bacteriocin produced by *Lactococcus lactis* subsp. *L.lactis* and has been directly used to preserve foods ranging from dairy to processed meats and fish to fruit juices as it is a prolific producer of bacteriocins (Juturu and Wu, 2018; Todorov et al., 2022; Field et al., 2023).

The well-studied bacteriocin known as enterocin AS-48, produced by *Enterococcus* sp., exhibits broad-spectrum antibacterial activity via dimer insertion into the plasma membrane to forms pore structures (Ross et al., 2020). Montalbán-López et al. discovered and successfully produced a linear form of the specific alpha-helical region of enterocin AS-48 that is directly responsible for the membrane-penetrating activity of the peptide (Montalbán-López et al., 2008). Our lab previously identified uncharacterized AS-48 homologs, which contained conserved similar membrane-interacting bioactive domains in *Bacillus safensis*, *Clostridium sordellii*, *Paenibacillus larvae*, and *Bacillus xiamenensis*. Using the truncated, linearized enterocin AS-48 and AS-48-like homologs from these four species, five peptide libraries have been generated using rational design techniques, each with 95 synthetic peptide variants and the respective parent peptide of each library (Fields et al., 2018; Ross et al., 2020). In a previous study, antibacterial activity screening of 384 of the peptides against gram-negative and gram-positive bacteria showed that 43 peptide variants outperformed their parent peptide scaffold, with two of the most potent peptide candidates exhibiting a minimum inhibitory concentration (MIC)—indicating the *in vitro* level of susceptibility or resistance of microbial strains to specific antimicrobial treatments—of 15.6 nM against BL21 *E. coli*, compared to the MIC of 32 µM of their parent scaffold peptide sequence (Ross et al., 2020; Kowalska-Krochmal and Dudek-Wicher, 2021). In another previous study by our lab, antileishmanial activity screening of all 480 peptides against the protozoan parasite *Leishmania donovani*, identified 172 peptide variants that exhibited 50% inhibitory concentration (IC_50_) values below 20 µM against the parasite, 60 peptide variants of which were shown to be inhibitory in the nanomolar range, against axenic amastigotes, with nine peptide variants exhibiting effective activity, meaning IC_50_ values below 4 µM, against intracellular amastigotes with limited *in vitro* host cell toxicity (Corman et al., 2022c).

Given these previous studies demonstrating the potential of these synthetic peptide libraries to exhibit significant activity against both eukaryotic and prokaryotic pathogens, we sought to investigate the utility of these potent peptides against the pathogens of a distinct kingdom—fungi. The decision to test for antifungal activity was motivated by the success of previous antifungal peptide studies as well as the demonstrated broad efficacy of our peptide libraries (Corman et al., 2022c; Madanchi et al., 2019; Ross et al., 2020; Zheng et al., 2021). The canon syn-enterocin peptide library previously generated by our lab using rational design techniques that have 95 synthetic peptide variants from the truncated, linearized parent enterocin AS-48 sequence, was screened against three clinically relevant fungal strains: *C. neoformans*, *C. albicans*, and *C. auris*. Twelve peptides exhibited significant antifungal activity against *C. neoformans*, and two peptides exhibited antifungal activity against *C. albicans*. None of these fourteen peptides showed cytotoxicity to an immortalized human keratinocyte cell line (HaCats). Four peptides were identified with minimum inhibitory concentrations (MICs) below 8 µM against *C. neoformans*, exceeding the effective MIC of the leading antifungal agent fluconazole. One of these four peptides, peptide no. 24, has previously been shown to be effective against gram-negative and gram-positive bacteria and another one of these peptides, peptide no. 19, has previously been shown to be effective against the parasite *L. donovani*. As a result of MIC assays demonstrating significant efficacy of peptides against only *C. neoformans*, subsequent tests were conducted only against *C. neoformans*. Fungistatic/fungicidal tests showed that three of the four peptides, nos. 24, 32, and 40, were demonstrably fungicidal. In 36-hour cell growth tests with these fungicidal peptides, peptide no. 32 exhibited *C. neoformans* cell counts slightly below those of the antifungal medication fluconazole. To gain a better understanding of the possible mechanism of activation of our antifungal peptide candidates, we screened peptide no. 32 against a whole *Cryptococcus* deletion library to identify possible targets for the designed peptides. Screening of peptide no. 32 against a deletion library of *C. neoformans* mutants revealed that the mechanism of action of peptide no. 32 may relate to multivesicular bodies (MVBs) or polysaccharide capsule targeting. The unique nature of the polysaccharide capsule of *C. neoformans* as well as the role of vesicular bodies in *C. neoformans* capsular polysaccharide synthesis and capsule assembly could explain the specificity of peptide no. 32 to *C. neoformans* (Crawford et al., 2020). These findings importantly demonstrate that naturally derived AMPs produced by bacteria can be sourced, engineered, and modified to exhibit potent antifungal activity. Our results will contribute to the development of broad treatment alternatives to fungal infections and lend themselves to direct implications for possible treatment options for *C. neoformans* infections.

## Materials and methods

### Rational design and synthesis of syn-enterocin peptide library

As previously described, the bioactive region of enterocin AS-48 was used as the scaffold for the syn-enterocin peptide library, while the bioactive regions of AS-48 homologs were used as the scaffolds for four other synthetic peptide libraries (Fields et al., 2018; Ross et al., 2020). Three specific approaches summarize the rational design techniques used to improve antimicrobial activity of these scaffolds (Ross et al., 2020). First, single amino acids were flipped step-wise to gradually improve amphipathicity (Fjell et al., 2012). Second, a short-chained amino acid was switched for lysine, intended to increase peptide affinity for anionic membranes (Morita et al., 2013; Jin et al., 2016). Third, aliphatic and non-polar short-chained amino acids were replaced with tryptophan, intended to improve phospholipid bilayer penetration (Fimland et al., 2002; Nguyen et al., 2010). Libraries were named after their parent peptide, hence syn(synthetic)-enterocin. All 95 of the 25-residue synthetic peptides of each library were commercially synthesized by GenScript (Piscataway, NJ, United States). Synthesis was confirmed to be >95% pure and verified by HPLC and mass spectrometry before use (GenScript). All peptides were suspended in Nanopure water and diluted to a final stock concentration of 1.28 mM. All peptides were stored at -20°C.

### Fungal culture

*C. neoformans* (KN99α) and *C. albicans* (SC5314) were grown at 30°C in yeast extract peptone dextrose (YPD) to an optical density (OD) between 0.5 and 1.2 at 600 nm. *C. auris* (B8441) was grown at 30°C in yeast nitrogen base (YNB) to an OD between 0.5 and 1.2 at 600 nm. All three yeasts were stored at -4°C. Stock plates of *C. neoformans* deletion mutants from the whole *Cryptococcus* deletion collection were kept at -80°C for long-term storage in YPD, nourseothricin (NAT), and 15-20% glycerol (obtained from Fungal Genetics Stock Center, Kansas State University). *C. neoformans* deletion mutants were thawed at 30°C for approximately 24 hours, diluted 1:20X in YPD, and grown to an OD between 0.5 and 1.2 at 600 nm.

### Antifungal peptide library screen and MIC determination

Antifungal peptide library screening and MIC determination were adapted from protocols used to screen the AS-48-based synthetic peptide antimicrobial libraries against bacteria (Ross et al., 2020). All 96 members of the syn-enterocin peptide library were screened at 16 µM in Roswell Park Memorial Institute (RPMI) media against *C. neoformans* (KN99α), *C. albicans* (SC5314), and *C. auris* (B8441) grown under the conditions previously described with approximately 500 fungal cells per well. *C. neoformans* and *C. auris* plates were incubated at 37°C and 5% CO_2_ for 96 hours after introduction of peptide and read at OD 600nm every 24 hours. *C. albicans* plates were incubated at 37°C without 5% CO_2_ for 96 hours after introduction of peptide and read at OD 600nm every 24 hours. The difference in CO_2_ was to prevent formation of *C. albicans* biofilms (Pentland et al., 2021). Peptide variants were determined to be potential positive hits if approximately 50% inhibition was observed, meaning that fungal growth as measured by OD was reduced 50% by peptide treatment relative to vehicle control. MICs were determined for suspected positive hits against *C. neoformans* and *C. albicans* grown under the conditions previously described with approximately 500 fungal cells per well. MIC assays were conducted in RPMI, and peptide concentrations ranged from 0.25 µM to 128 µM. *C. neoformans* plates were incubated at 37°C and 5% CO_2_ for 96 hours after introduction of peptide and read at OD 600nm every 24 hours. *C. albicans* plates were incubated at 37°C without 5% CO_2_ for 96 hours after introduction of peptide and read at OD 600nm every 24 hours. MICs were determined by approximately 50% inhibition of fungal growth, meaning that fungal growth as measured by OD was reduced 50% by peptide treatment relative to vehicle control.

### Cytotoxicity against HaCat cells

Cytotoxicity assays against an immortalized human keratinocyte cell line (HaCaT) were conducted under the same conditions as in previous protocols used to analyze the cytotoxicity of members of the AS-48-based synthetic peptide antimicrobial libraries (Ross et al., 2020). Briefly, HaCaT cells were grown to 80% confluency in 24-well culture dishes, treated, and incubated with 4 μM ethidium homodimer (Molecular Probes) for 30 min at room temperature. Fluorescence was measured using the Synergy H1 Microplate Reader set to 528 nm excitation, 617 nm emission, and a cutoff value of 590 nm. Saponin (0.1% wt/vol, Sigma) was added to each well followed by a covered incubation for 20 min at room temperature, followed by another measurement on the Synergy H1 Microplate Reader using the same settings to normalize readings to the number of cells per well and determine the percent cytotoxicity. Peptides were characterized as cytotoxic at a particular concentration if incubation with that concentration of peptide resulted in more than 20% cell death after 16 h, which corresponded to the percent cytotoxicity in HaCaTs treated with the vehicle control under the same conditions.

### Fungicidal/fungistatic tests

Fungicidal/fungistatic tests were conducted with the four especially effective syn-enterocin peptides. *C. neoformans* was grown at 30°C in YPD overnight to an OD between 0.5 and 1.2 at 600 nm, then the *C. neoformans* culture was diluted to 10^5^ and 10^3^ fungal cells per well on a 96-well plate. Each well was treated with peptide at 16 µM before 5 µL per well was transferred onto YPD agar plates. Dilutions to reach desired cell counts were done in YPD. Six replicates of each peptide/dilution combination were plated. Plates were incubated at 30°C for 72 hours. Fungal growth was determined by visual inspection, with presence or absence of fungal growth being recorded (individual colonies were absent or overgrown and thus uncountable).

### Cell count growth curves

Cell count growth curves were generated for the three peptides fungicidal against *C. neoformans*. Peptides were screened at 16 µM at 30°C against 300,000 fungal cells in 600 µL of YPD. Cells were counted at 0, 6, 12, 24, 30, and 36 hours using a TC20 Automated Cell Counter with default settings (Bio-Rad). This automated cell counter used microscopy with auto-focus to analyze multiple and select the best focal plane, using its cell counting algorithm and images from the best focal plane to calculate total cell count while excluding debris.

### *C. neoformans* mutant library screening

Peptide no. 32 was screened against all 4,701 *C. neoformans* deletion mutants from the whole *Cryptococcus* deletion collection under the same conditions as antifungal peptide screening. (obtained from Fungal Genetics Stock Center, Kansas State University). Deletion 96-well plates were stored at -80°C, then thawed for at least two hours at 30°C. *C. neoformans* deletion mutants were diluted in RPMI to approximately 500 fungal cells per well before being treated with peptide no. 32 at 16 µM. *C. neoformans* plates were incubated at 37°C and 5% CO_2_ for 96 hours after introduction of peptide and read at OD 600nm every 24 hours. Top deletion mutants with growth, measured by OD relative to the average OD of sterility wells, were suspected of resistance to peptide treatment. Suspected hits were confirmed with six replicates.

## Results

### Peptide library screen and MIC determination

**Figure 1:**
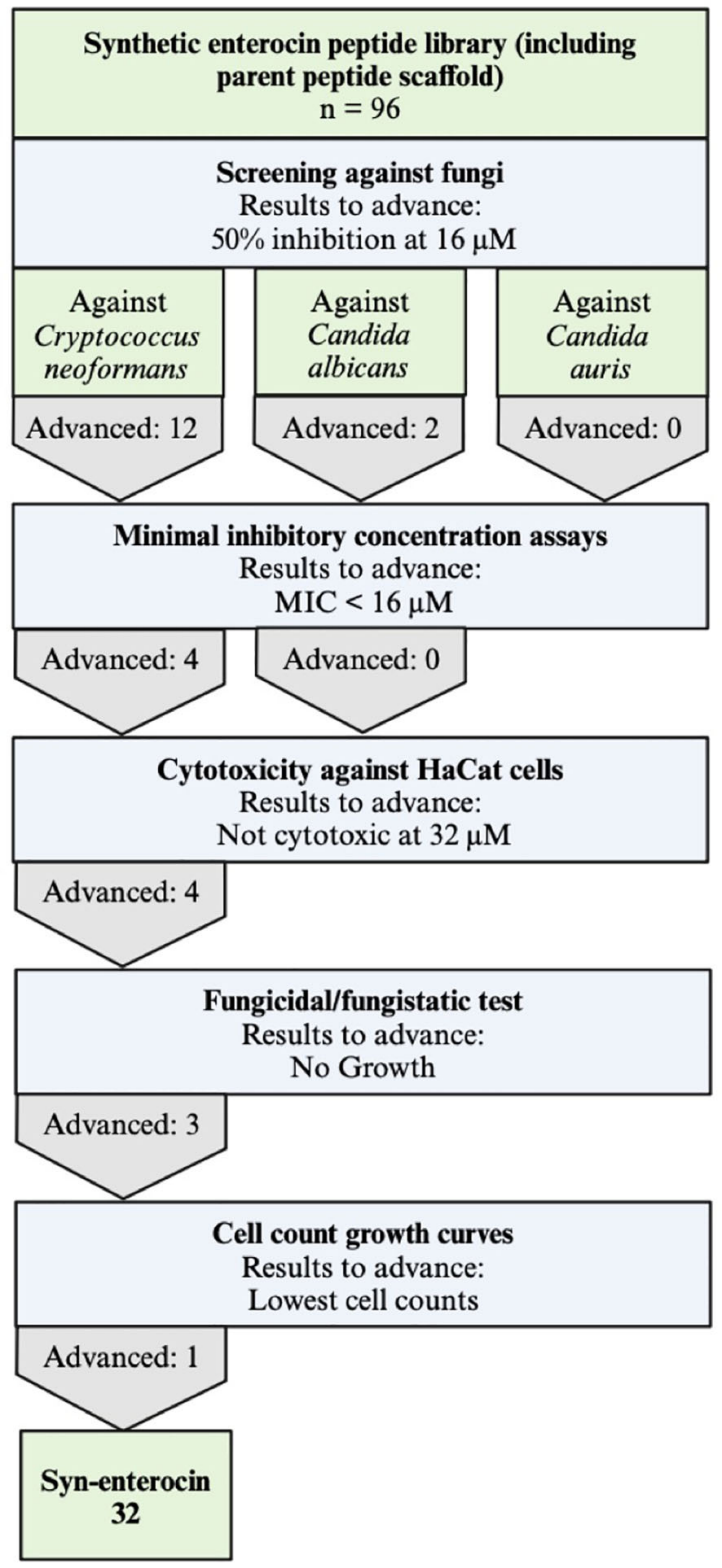
Experiemental workflow to determine potent fungicidal peptides. All 96 members of the synthetic enterocin peptide library, including the parent peptide scaffold, were screened against *C. neoformans, C. albicans*, and *C. auris* and advanced to the next round if they had approximately 50% inhibition observed at 16 μM. After minimal inhibitory concentration (MIC) assays, only peptides with MICs lower than 16 μM advanced to the next round, resulting in further tests exclusively against *C. neoformans*. HaCat cells were used to determine cytotoxicity, and peptides not cytotoxic at 32 μM advanced. Of the three peptides determined to be fungicidal, peptide no. 32 had the lowest cell counts in cell growth curves.

In this study, we sought to investigate the fungicidal utility of minimal domain active peptides derived from a rationally designed library of bacteriocin-based peptides. The syn-enterocin peptide library previously generated by our lab using rational design techniques has 95 synthetic peptide variants from the truncated, linearized parent enterocin AS-48 sequence. These peptides were tested for antifungal activity against three clinically relevant fungal strains: *C. neoformans*, *C. albicans*, and *C. auris*. All 96 peptide variants of the syn(synthetic)-enterocin, including the AS-48 domain parent peptide, were screened against *C. neoformans* (KN99α), *C. albicans* (SC5314), and *C. auris* (B8441) at 16 µM. Peptide variants were determined to be potential positive hits if inhibition was observed during screening at 16 µM, meaning that fungal growth as measured by OD was reduced by peptide treatment relative to vehicle control. Twelve peptides were found to be potential positive hits against *C. neoformans*, and two peptides were found to be potential positive hits against *C. albicans*. None of the 96 screened peptides exhibited inhibitory activity against *C. auris*. Potential positive hits were confirmed by subsequent minimal inhibitory concentration (MIC) assays (**Table 1**, **Supplementary Figure 1**). MIC assays confirmed eight peptides as positive hits against *C. neoformans* at 16 µM, with four peptides being especially effective, meaning those peptides had MICs of 8 µM or lower. Notably, syn-enterocin peptide no. 32, sequence AGWERIAKELKKYIKKGKKVIRAAW, exhibited an MIC of 2 µM against *C. neoformans*. MIC assays did not confirm positive hits against *C. albicans*. As a result of there being no confirmed peptide hits against either *Candida* species, all subsequent tests were performed with *C. neoformans* (**Figure 1**).

**Table 1:** MICs of potential positive hits.

Against C. *neoformans*		Against C. *albicans*	
Peptide variant	MIC (μM)	Peptide variant	MIC (μM)
11	32	51	>128
14	16	55	>128
15	16		
19	8		
20	32		
24	8		
32	2		
40	8		
46	16		
50	64		
51	32		
56	16		

### Cytotoxicity against HaCat cells

As a preliminary evaluation of clinical relevance, the four selected effective peptides with MICs of 8 µM or lower were screened for cytotoxicity against human keratinocyte cells (HaCats). Cytotoxicity against HaCats was determined via membrane permeabilization using ethidium homodimer assays. None of the four especially effective peptides were cytotoxic at their respective MIC against *C. neoformans* or even 32 µM (data not shown). Peptides were screened at concentrations from 0.25 µM to 128 µM. These data are consistent with previous studies showing that our synthetic peptide library from an AS-48 domain parent peptide has low eukaryotic toxicity (Ross et al., 2020).

### Fungicidal/fungistatic tests

**Figure 2:**
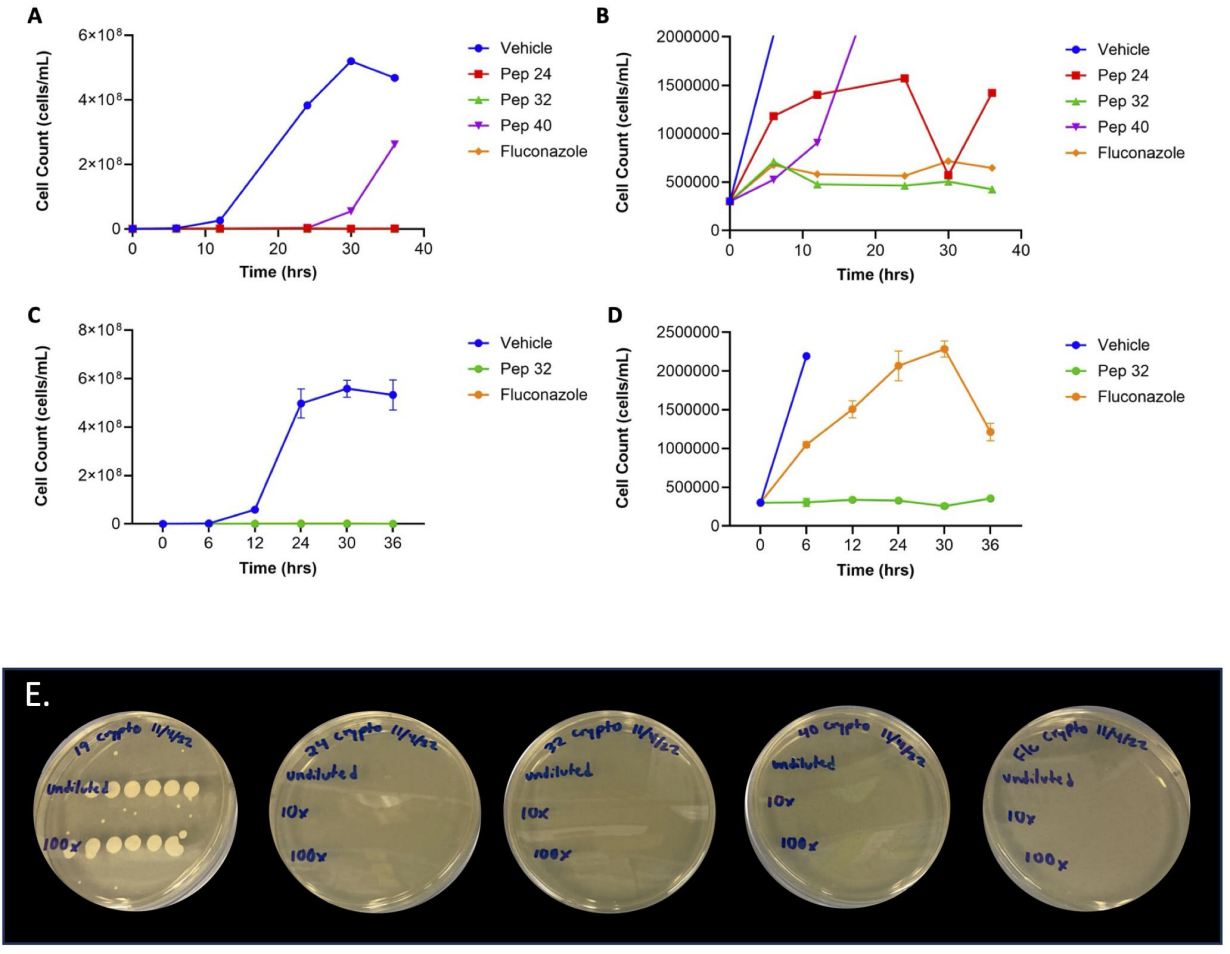
Cell count growth curves of fungicidal peptides. **(A)** At 16 μM, all three fungicidal peptides had cell counts well-below those of vehicle, with the cell counts of peptides 24 and 32 not being visible on the scale of the cell counts of vehicle. **(B)** The bottom of the graph in **Figure 2A** showed that peptide 32 had lower cell counts than both peptide 24 and fluconazole. **(C)** The low cell counts of peptide 32 were confirmed in triplicate, shown well-below those of vehicle. **(D)** The bottom of the graph in **Figure 2C** confirmed that the cell counts of peptide 32 were lower than those of fluconazole. **(E)** Fungicidal/fungistatic testing of select peptides. Treatment with peptide no. 19 resulted in extensive fungal growth, with only 10^3^ *C. neoformans* cells, suggesting that peptide no.19 has a fungistatic effect on *C. neoformans*. Treatment with peptide no. 24 resulted in no fungal colonies after 72 hours, even with 10^5^ *C. neoformans* cells, suggesting that peptide no. 24 has a fungicidal effect on *C. neoformans*. Similarly, treatment with peptide no. 32 resulted in no fungal colonies, even with 10^5^ *C. neoformans* cells, suggesting that peptide no. 32 has a fungicidal effect on *C. neoformans*. Treatment with peptide no. 40 also resulted in no *C. neoformans* colonies, even 10^5^ fungal cells, suggesting a fungicidal effect of peptide no. 40 on *C. neoformans*. Fluconazole was used as an *in vitro* fungicidal control against *C. neoformans*. Initial cell count before treatment is given in the leftmost column. All treatments were at 16 μM concentration.

To assess which of the effective peptides are fungicidal and which are fungistatic, all four effective peptides were separately plated with *C. neoformans* on nutrient rich agar plates. Under these conditions, after 72 hours, treatment with peptide no. 19 had no effect on *C. neoformans* growth, with extensive fungal growth that prevented quantifying individual colonies (**Table 2**, **Figure 2E**). Thus, the relatively low MIC of peptide no. 19 likely represented a fungistatic effect of peptide no. 19 on *C. neoformans*. Treatments with peptide nos. 24, 32, and 40 each resulted in no fungal growth at 72 hours, even when with 10 (Mina et al., 2022) C*. neoformans* cells (**Table 2**, **Figure 2E**). These results demonstrated that three members of the syn-enterocin peptide library have both relatively low MICs and fungicidal activity against *C. neoformans*.

**Table 2:** Presence (+) or absence (–) of fungal growth at 72 hrs following treatment with fungistatic or fungicidal peptides.

	Treatment (16 μM)				
Cell Count	Peptide 19	Peptide 24	Peptide 32	Peptide 40	Fluconazole
10^5^	+	–	–	–	–
10^3^	+	–	–	–	–

### Cell count growth curves

To compare the relative efficacies of the three fungicidal peptides, cell count growth curves were generated over 36 hours. Initial cell count growth curves showed that independent treatment with all three fungicidal peptides at 16 µM resulted in fewer *C. neoformans* cells compared to vehicle (**Figure 2A**). These growth curves also suggested that treatment with peptide no. 32 resulted in fewer *C. neoformans* cells compared to treatment with fluconazole (**Figure 2B**). The efficacy of peptide no. 32 relative to fluconazole was corroborated by further cell count growth curves done in triplicate (**Figures 2C**, **D**). Taken together, these data confirm the high efficacy of peptide no. 32 against *C. neoformans* from the 96-member syn-enterocin library comparable to fluconazole.

### Secondary structure and thermostability analyses

**Figure 3:**
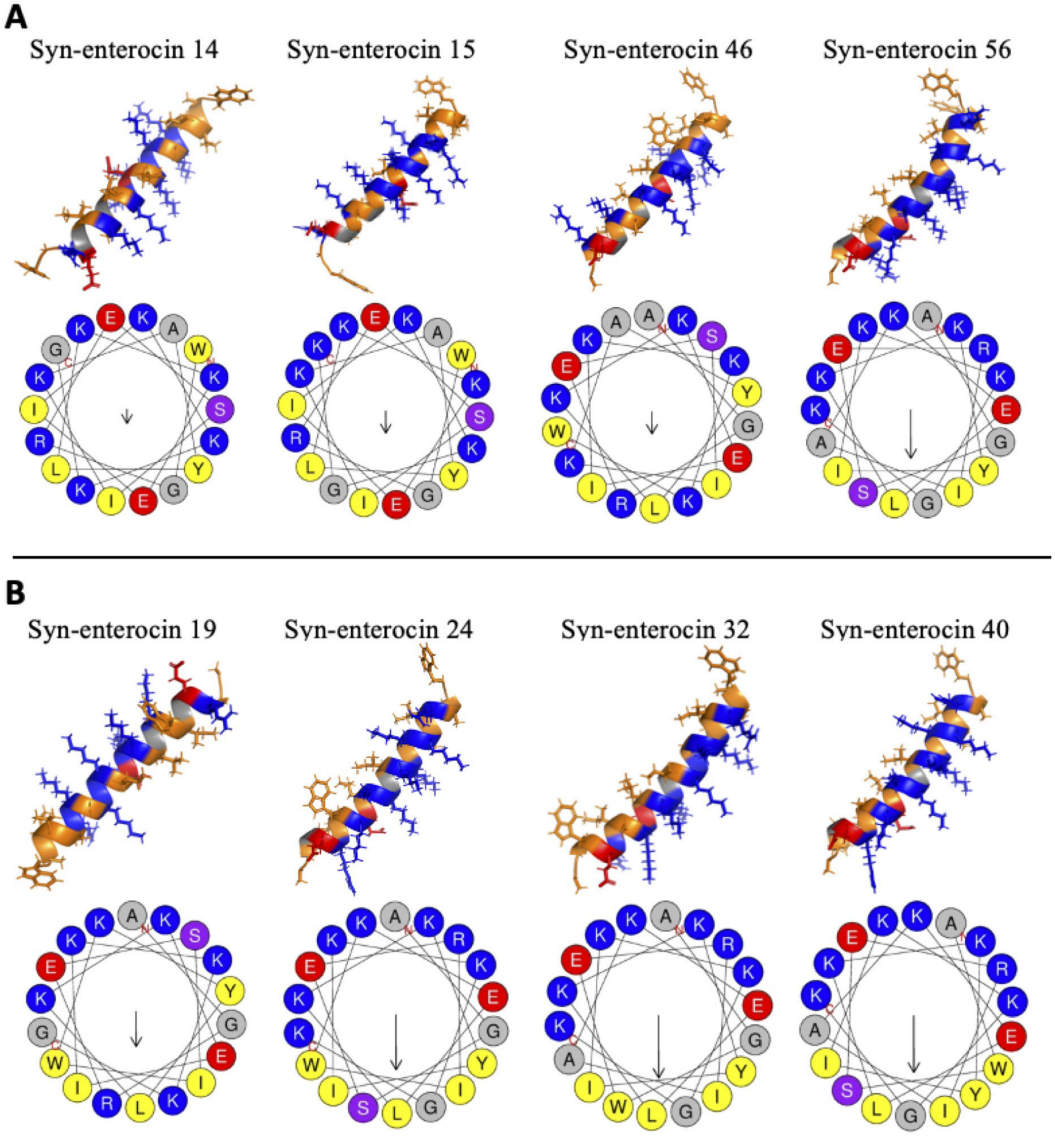
Secondary structure predictions for positive peptide hits. Secondary structures were modeled using AlphaFold and visualized in PyMOL, with orange hydrophobic regions, blue positive regions, and red negative regions. Helical wheels were predicted using HeliQuest, with relative hydrophobic moment indicated by arrow size. **(A)** The secondary structures and helical wheels of positive hits with MICs of 16 μM were grouped for ease of comparison. **(B)** The secondary structures and helical wheels of positive hits with MICs below 16 μM were also grouped for ease of comparison.

Predictions of the secondary structures of the positive peptide hits against *C. neoformans* showed that all eight of the positive hits exhibited a strong α-helical structure (**Figure 3**). Of the peptides with MICs of 16 µM, three had minimal curvature in their α-helix, while one had a noticeable curve (**Figure 3A**). Conversely, of the peptides with MICs below 16 µM, one had minimal curvature, while three have a noticeable curve (**Figure 3B**). Intriguingly, among those peptides with MICs below 16 µM, the one without a slight curve was fungistatic, while the three, including peptide no. 32 with the lowest MIC of all syn-enterocin peptides, with a slight curve were fungicidal. The slightly curved peptides, particularly the three fungicidal peptides, appear to have the majority or all of their positively charged residues (visualized in blue) on the convex side of their α-helix and the majority or all of their hydrophobic residues on the concave side of their α-helix. This distinction in curvature could contribute to differences in efficacy and lethality.

Of the eight positive hits, the four peptides with noticeable curvature in their α-helix had the highest hydrophobic moments (µH), with the three fungicidal peptides having the three highest (**Figure 3**, **Table 3**). Peptide no. 32, the most effective peptide, had the highest hydrophobic moment (µH = 0.625). These predictions suggest an integral role of hydrophobic moment in peptide effectiveness against *C. neoformans*. Interestingly, from a synthetic library of varied net charge (z), all eight positive hits had the same net charge (z = 5). The hydrophobicity (H) of the positive hits varied slightly from -0.077 to 0.050, with peptide no. 32 having the highest predicted hydrophobicity value. Predicted melting temperature was used to assess peptide thermostability. Melting temperatures of the positive hits varied from 54.3°C to 64.3°C, with peptide no. 32 having the second highest melting temperature (62.9°C).

**Table 3:** Biochemical characterization and stability analysis of positive peptide hits.

Peptide variant	z	H	*μ*H	Melting temp (°C)
14	5	0.031	0.152	56.7
15	5	0.031	0.235	55.9
19	5	0.031	0.364	54.3
24	5	0.031	0.557	59.6
32	5	0.050	0.625	62.9
40	5	0.048	0.566	56.4
46	5	0.048	0.227	55.6
56	5	-0.077	0.511	64.3

### Screening of *C. neoformans* mutant library to identify mechanism of action

**Figure 4:**
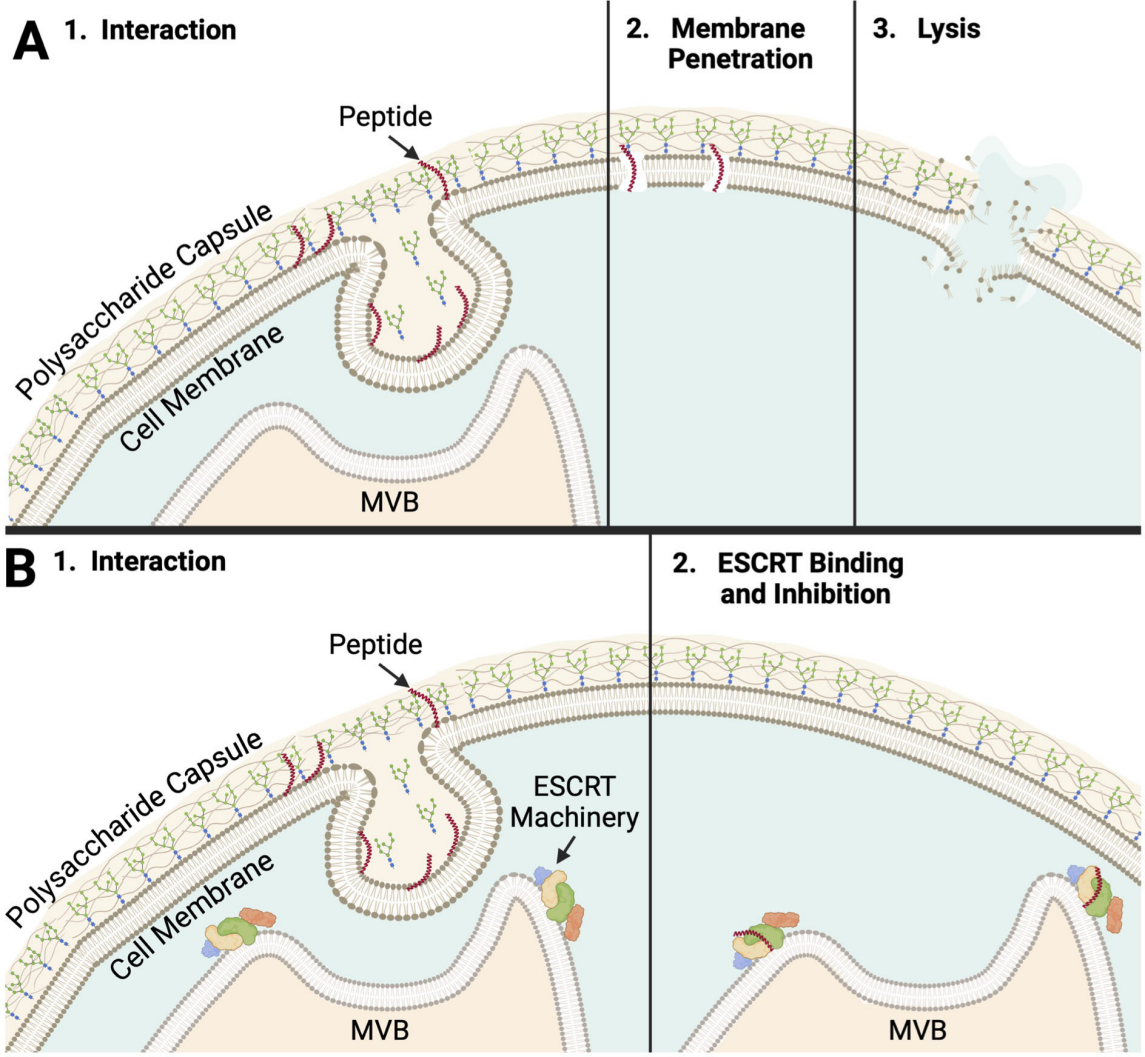
Peptide No. 32 Potential Modes of Action **(A)** 1. The peptide must first interact with the polysaccharide capsule of *C. neoformans.* In one theoretical mode of action, peptide no. 32 interacts with or binds a target on the cell surface. Targets could include structures related to MVBs, polysaccharide capsule formation, or both. 2. Peptide no. 32 like, the membrane-penetrating peptide AS-48 it was designed from, could penetrate the cell membrane, beginning to form pores. 3. The compromised cell membrane causes lysis and cell death, consistent with the fungicidal nature of peptide no. 32. **(B)** 1. In another theoretical mode of action, peptide no. 32, a short peptide, maybe able to pass through the polysaccharide capsule, cell wall, and cell membrane following interaction. Peptide no. 32 may only be able to do this during specific opportunities such as events during vesicular export of capsular polysaccharides. 2. Peptide no. 32 could bind a configuration of ESCRT machinery or a target related to the ESCRT pathway unique to *C. neoformans.* Resultant inhibition of MVB regulation could lead to cell death. Created with BioRender (Cohen, 2024).

As peptide no. 32 achieved significantly lower *C. neoformans* cell counts at 16 µM than all other peptide candidates from the syn-enterocin library and fluconazole, the mechanism of action of peptide no. 32 was the most consequential mechanism to investigate. Thus, peptide no. 32 was screened against a 4,701-member *C. neoformans* deletion library to gain information on the possible mechanism of antifungal action of peptide 32. Thirteen members of the deletion library were confirmed to be resistant to peptide no. 32 at 16 µM (**Supplementary Figure 2**, **Table 4**). The function of nine of the deleted genes was available on FungiDB. Intriguingly, the functions of four—three ESCRT complex subunits and a protein in MVBs—of these nine known genes relate closely to MVBs (Schmidt and Teis, 2012). Another known gene, CPS1, functions to synthesize the polysaccharide hyaluronic acid, a structural unit of the characteristic *C. neoformans* capsule (Jong et al., 2007). The structures produced by the regular expression of these genes possibly affect peptide no. 32 interaction prior to membrane penetration and lysis (**Figure 4A**). Such a mode of action could explain the specificity of peptide no. 32 to *C. neoformans* and inactivity against fungal strains with different cell surfaces such as *Candida* species. Alternatively, peptide no. 32 may bind an intracellular target such as ESCRT machinery, with the specificity of peptide no. 32 against *C. neoformans* potentially resulting from unique cryptococcal MVB regulation of the polysaccharide capsule (**Figure 4B**).

**Table 4:** C*. neoformans* gene deletions conferring resistance to peptide no. 32.

CNAG	Gene name	Function
CNAG_05520		hypothetical protein
CNAG_02125		hypothetical protein
CNAG_05431	RIM101	pH-response transcription factor pacC/RIM101
CNAG_04863	VPS25	ESCRT-II complex subunit VPS25
CNAG_00248		ESCRT-II complex subunit VPS36
CNAG_01720	VPS23	ESCRT-I complex subunit TSG101
CNAG_01862		hexose transporter
CNAG_07474		AP-2 complex subunit mu-1
CNAG_04320	CPS1	polysaccharide synthase Cps1p
CNAG_00583		hypothetical protein
CNAG_00613	FCY1	cytosine deaminase
CNAG_01193		hypothetical protein
CNAG_01265		charged multivesicular body protein 6

Intriguingly, peptide no. 32 was effective against *C. neoformans* cells with polysaccharide capsule, such as the wildtype strain, as well as against members of the deletion library known to lack a polysaccharide capsule, such as *CAP60* and *CAP64* (Grijpstra et al., 2009). One explanation of this activity against capsular and acapsular phenotypes is that both capsular wildtype strains and acapsular *CAP* mutants secrete polysaccharides, potentially providing the same MVB-related opportunities for the interaction and binding of peptide no. 32 (Grijpstra et al., 2009). A phenotype between regular and absent capsules could confer resistance to peptide no. 32 as mutants with deletions of genes such as polysaccharide synthases like *CPS1* or ESCRT complex subunits like *VPS23* confer a reduced capsule phenotype in *C. neoformans* (Chang et al., 2006; Hu et al., 2013). Some aspect of the this disrupted but not totally reduced capsular phenotype lacks the target or cellular event unique to *C. neoformans* that is required for the activity of peptide no. 32. Taken together, these results reveal insights into fungal, and especially cryptococcal, cell biology and thus into crucial targets for anticryptococcal treatments.

## Discussion

In this paper, we determined the most effective antifungal peptide from a synthetic enterocin peptide library and interrogated its mechanism of action. From initial screening and subsequent MIC assays, it was determined that several members of the library were effective at reducing *C. neoformans* concentrations and that no members of the library were effective at reducing *C. albicans* or *C. auris* concentrations. Cytotoxicity assays against HaCat cells further revealed the specificity of the most effective peptides among eukaryotic cells as none of the peptides tested against the HaCats were cytotoxic against HaCats. Three of the most effective peptides were determined to be fungicidal against *C. neoformans* in nutrient-rich media. Cell count growth curves conveyed that at 16 µM, syn-enterocin peptide no. 32, sequence AGWERIAKELKKYIKKGKKVIRAAW, is the most effective member of the library against *C. neoformans* and even more effective than fluconazole, the well-established first-line clinical antifungal (Kowalsky and Dixon, 1991; Setianingrum et al., 2019).

Peptide no. 32 having been determined to be the most effective member of the library, it followed that the specific mechanism of action of peptide no. 32 should be investigated. The resistant members of the whole *Cryptococcus* deletion collection revealed several possible targets for activity of peptide no. 32. The most prevalent targets, suggested by four of the nine known deletions that conferred resistance, related to MVBs, either directly in the case of charged multivesicular body protein 6 (CHMP6) or indirectly in the cases of the three ESCRT subunits—ESCRT-I complex subunit VPS23/TSG101, ESCRT-II complex subunit VPS25, and ESCRT-II complex subunit VPS36 (Schmidt and Teis, 2012). Peptide no. 32 having activity on targets related to MVBs aligns well with the membrane-penetrating activity of the natural and linearized forms of AS-48 that peptide no. 32 is derived from (Montalbán-López et al., 2008; Ross et al., 2020). These results also align well with the α-helical structures predicted by PEPFOLD3. Subunits of both ESCRT-I and ESCRT-II being targets of the peptide could make sense because it is theorized that ESCRT-I and ESCRT-II operate at the same step of ubiquitinated-cargo recognition and sorting as part of a supercomplex (Piper and Katzmann, 2007; Henne et al., 2011). Further, biochemical analysis showing ESCRT-I and ESCRT-II to be distinct complexes suggests that an ESCRT-I and ESCRT-II supercomplex forms on membranes, the intended sites of syn-enterocin peptide insertion (Piper and Katzmann, 2007; Henne et al., 2011). Subunits or products of ESCRT-I and ESCRT-II specifically being targeted by peptide no. 32 would align with the specificity of peptide no. 32 to *C. neoformans* and not bacteria, *Leishmania donovani*, or HaCats as bacteria lack ESCRT-0, -I, and -II components and many eukaryotes such as humans may have different forms of the ESCRT-II subunits than the ones identified in *C. neoformans* (Junglas et al., 2021; Liu et al., 2021; Olmos, 2022).

Another potential target of peptide no. 32 identified by deletion mutant screening was polysaccharide synthase Cps1p. The *CPS1* gene encodes hyaluronic acid synthase and is related to the formation of the polysaccharide capsule of *C. neoformans*, a hallmark factor required for *C. neoformans* virulence (Chang et al., 2006; Jong et al., 2007; Casadevall et al., 2019). This potential target could help to explain the specificity of peptide no. 32 as the formation of a polysaccharide capsule is unique to *C. neoformans* and not shared by the other fungal species such as those that peptide no. 32 was screened against, *C. albicans* and *C. auris*. The polysaccharide capsule surrounding *C. neoformans* cells is 90-95% glucuronoxylomannan (GXM), which has a negative charge and contributes to the electrostatic forces that help C. neoformans cells resist phagocytosis (Rathore et al., 2022). The syn-enterocin peptide library was specifically designed anionic affinity, likely contributing to the ability of peptide no. 32, with a net charge of 5, to interact with the negatively charged capsule and membrane of C. neoformans (**Table 3**). As the deletion of *CPS1* results in a smaller polysaccharide capsule and decreased, *C. neoformans* strains that gain resistance to a treatment targeting polysaccharide synthase Cps1p may have concomitant and potentially impactful decreases in virulence (Chang et al., 2006). Intriguingly, peptide no. 32 was effective against members of the deletion library that lack a polysaccharide capsule such as mutants with deletions of *CAP60* and *CAP64* (Grijpstra et al., 2009). At least two of the deletions conferring resistance to peptide no. 32, namely deletions of *CPS1* or *VPS23*, are known to confer reduced polysaccharide capsules (Chang et al., 2006; Hu et al., 2013).

The current understanding of the *C. neoformans* capsule assembly may provide harmony between the seemingly unrelated potential targets of peptide no. 32. Capsular polysaccharides are synthesized within vesicular bodies, and export of those vesicular bodies is necessary for the *C. neoformans* polysaccharide capsule to be assembled (Crawford et al., 2020). While the mechanisms of capsule assembly in *C. neoformans* require deeper exploration, the relationship between MVBs and capsule formation could suggest a key event during capsule assembly that produces a target, or targets, for peptide no. 32 that is removed when certain MVB or capsule synthesis genes are deleted (**Figures 4A, B**). That target may not be removed in acapsular C. neoformans mutants such as CAP60 and CAP64 depending on the mechanisms by which polysaccharides are still secreted without forming a polysaccharide capsule (Grijpstra et al., 2009). Further, the established membrane-penetrating abilities of AS-48-like peptides suggest that peptide no. 32 too could insert into the plasma membrane of *C. neoformans* cells, form pores, and cause lysis (**Figure 4A**). Alternatively, peptide no. 32 could have an intracellular target such as ESCRT machinery that it binds, inhibiting MVB regulation and eventually causing cell death (**Figure 4B**). Peptide interaction during a unique event such as capsular polysaccharide synthesis, polysaccharide secretion, or capsule assembly would explain the specificity of peptide no. 32 activity against *C. neoformans* as well as its inactivity against *Candida* species.

These results can be applied to several developing areas of research such as synthetic peptide design, treatment of fungal infections, and fungal cell biology. These results add fungi to the list of pathogens that AS-48-based synthetic peptide libraries have been tested against in addition to several bacterial species and *Leishmania donovani*, allowing for broader analysis of the overall antimicrobial efficacy of libraries. The pursuit of the specific application of peptide no. 32 to *C. neoformans* infections would logically proceed to *in vivo* studies, potentially followed by clinical trials. These results also lend themselves to the potential future use of fungistatic and fungicidal peptides synergistically with existing antifungals to reduce overall MICs and development of resistance.

## Data Availability

The original contributions presented in the study are included in the article/**Supplementary Material**. Further inquiries can be directed to the corresponding author.
