# Supplementary material for: Minimal domain peptides derived from enterocins exhibit potent antifungal activity
Source: Front Fungal Biol. 2024 Dec 19;5:1506315. doi: 10.3389/ffunb.2024.1506315 (PMC11693670; doi:10.3389/ffunb.2024.1506315)
Supplement: Supplementary file 1 [file DataSheet1.pdf]

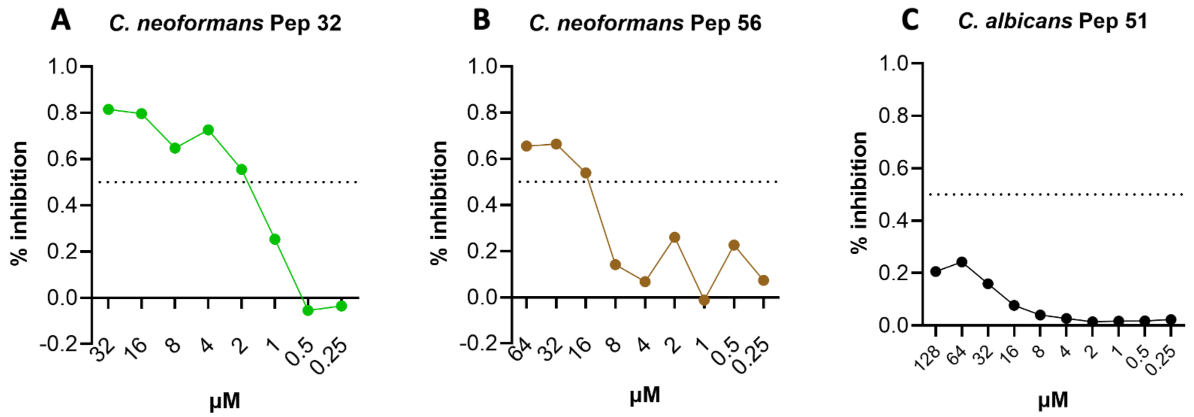

Supplemental Figure 1. Representative MIC plots of potential positive hits. **A.** The MIC plot of peptide no. 32 against *C. neoformans* showed that treatment with concentrations of peptide no. 32 at 2 µM and above results in 50% or greater inhibition of *C. neoformans* growth. Thus, the MIC of peptide no. 32 against *C. neoformans* was 2 µM. **B.** The MIC plot of peptide no. 56 against *C. neoformans* showed that treatment with concentrations of peptide no. 56 at 16 µM and above resulted in 50% or greater inhibition of *C. neoformans* growth. The MIC plot of peptide no. 51 against *C. albicans* showed that treatment with concentrations of peptide no. 51 of up to 128 µM resulted in well below 50% inhibition of *C. albicans* growth. Thus, the MIC of peptide no. 51 against *C. albicans* was above 128 µM, the highest peptide concentration tested. If treatment with peptide concentrations of 32 µM or 64 µM demonstrated inhibition of 50% or higher, higher concentrations were not tested. Percent inhibition was calculated as the reduction in OD at 600nm following peptide treatment relative to vehicle control. The average of six replicates was used for each peptide concentration treatment and vehicle control.
