# Supplementary material for: Minimal domain peptides derived from enterocins exhibit potent antifungal activity
Source: Front Fungal Biol. 2024 Dec 19;5:1506315. doi: 10.3389/ffunb.2024.1506315 (PMC11693670; doi:10.3389/ffunb.2024.1506315)
Supplement: Supplementary file 2 [file DataSheet2.pdf]

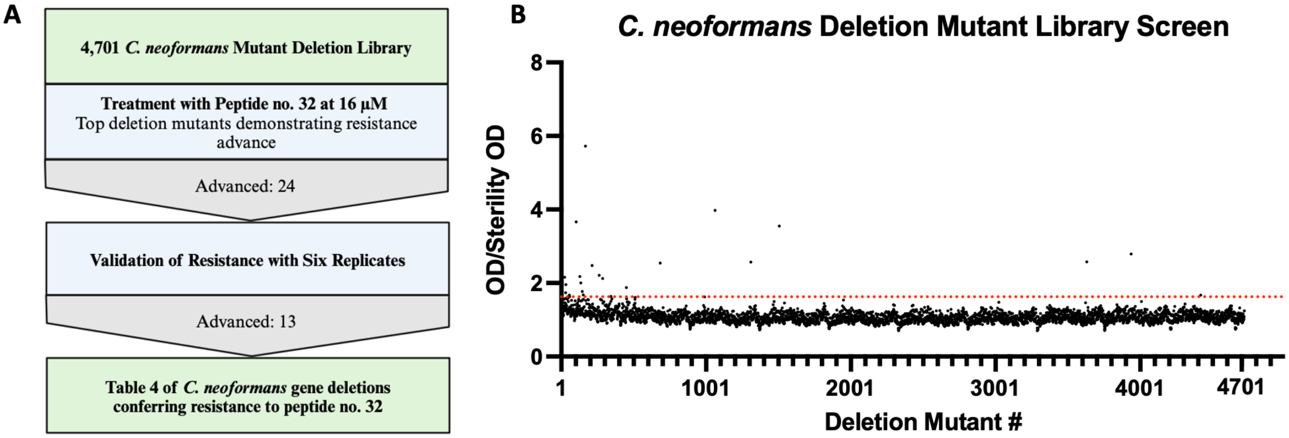

Supplemental Figure 2. *C. neoformans* Deletion Mutant Library Screen. **A.** The experimental workflow shows how the gene deletions conferring resistance to peptide no. 32 at 16 µM were determined. The twenty-four *C. neoformans* deletion mutants with growth suggesting resistance, measured by ODs well above the average ODs of sterility wells of each plate, advanced from the initial screen. Thirteen of the twenty-four suspected hits were validated with six replicates. **B.** After 96 hours, almost every *C. neoformans* mutant well treated with peptide no. 32 at 16 µM had an OD approximately the same as that of the average sterility well without any fungal cells. The twenty-four deletion mutants that exhibited resistance to peptide no. 32 with the highest ODs relative to sterility were tested further.
